# Genotype-Temperature Interaction in the Regulation of Development, Growth, and Morphometrics in Wild-Type, and Growth-Hormone Transgenic Coho Salmon

**DOI:** 10.1371/journal.pone.0009980

**Published:** 2010-04-01

**Authors:** Mare Lõhmus, L. Fredrik Sundström, Mats Björklund, Robert H. Devlin

**Affiliations:** 1 Centre for Aquaculture and Environmental Research, Fisheries and Oceans Canada, West Vancouver, British Columbia, Canada; 2 Section for Environment and Biosecurity, Department for Chemistry, Environment and Feed Hygiene, National Veterinary Institute, Uppsala, Sweden; 3 Animal Ecology/Department of Ecology and Evolution, Evolutionary Biology Centre, Uppsala University, Uppsala, Sweden; University of Alabama, United States of America

## Abstract

**Background:**

The neuroendocrine system is an important modulator of phenotype, directing cellular genetic responses to external cues such as temperature. Behavioural and physiological processes in poikilothermic organisms (e.g. most fishes), are particularly influenced by surrounding temperatures.

**Methodology/Principal Findings:**

By comparing the development and growth of two genotypes of coho salmon (wild-type and transgenic with greatly enhanced growth hormone production) at six different temperatures, ranging between 8° and 18°C, we observed a genotype-temperature interaction and possible trend in directed neuroendocrine selection. Differences in growth patterns of the two genotypes were compared by using mathematical models, and morphometric analyses of juvenile salmon were performed to detect differences in body shape. The maximum hatching and alevin survival rates of both genotypes occurred at 12°C. At lower temperatures, eggs containing embryos with enhanced GH production hatched after a shorter incubation period than wild-type eggs, but this difference was not apparent at and above 16°C. GH transgenesis led to lower body weights at the time when the yolk sack was completely absorbed compared to the wild genotype. The growth of juvenile GH-enhanced salmon was to a greater extent stimulated by higher temperatures than the growth of the wild-type. Increased GH production significantly influenced the shape of the salmon growth curves.

**Conclusions:**

Growth hormone overexpression by transgenesis is able to stimulate the growth of coho salmon over a wide range of temperatures. Temperature was found to affect growth rate, survival, and body morphology between GH transgenic and wild genotype coho salmon, and differential responses to temperature observed between the genotypes suggests they would experience different selective forces should they ever enter natural ecosystems. Thus, GH transgenic fish would be expected to differentially respond and adapt to shifts in environmental conditions compared with wild type, influencing their ability to survive and interact in ecosystems. Understanding these relationships would assist environmental risk assessments evaluating potential ecological effects.

## Introduction

Growth of organisms arises as a net outcome of numerous behavioural and physiological functions and is influenced by variables such as food intake, digestion, absorption, and assimilation, as well as metabolic expenditure and excretion. The physiological limitations to growth are in turn strongly influenced by both biotic factors, such as the size of the individual and the availability of nutrition, and by abiotic factors, such as day length and temperature [1].

Poikilotherms, such as most fish, have limited biological means to control and regulate body temperature. Consequently they are particularly affected by thermal conditions which influence their metabolic rate and oxygen consumption, growth and other physiological characteristics [2], [3], [4], [5]. Since ambient temperatures vary both daily and seasonally, poikilotherms are often within a temperature regime that is not optimal for all functions. Hence these organisms have developed mechanisms to survive various thermal conditions both above and below optimal ranges [3], [6].

The nervous and endocrine systems are major signalling pathways between external cues, such as the ambient temperature, and internal physiology responding to environmental changes [7]. Hormones, the chemical messengers of the endocrine system, exert profound effects on organisms' physiology and behaviour and are able to act simultaneously on many target tissues, including the brain [8]. Thus, it is not surprising that the neuroendocrine machinery, more than any other physiological system, is critically involved in the evolution of entire suites of complex adaptive traits [9]. In general, hormonal control systems are rather complicated, including multiple levels of hierarchical control, negative and positive feedback loops and numerous signal substances. Consequently, an alteration in the secretion of one hormone not only affects the parameters of one control system but also others, and can have pleiotropic effects on many bodily functions [10].

Growth hormone (GH) is a principal regulator of somatic growth in vertebrates, being produced in the pituitary gland and regulated by neuroendocrine controls integrating external environmental (e.g. increased daylength and temperature in the spring) and internal metabolic (e.g. energy status) signals. GH has major metabolic effects on lipid mobilization and protein accretion, increases gluconeogenesis, and enhances feed intake and conversion during growth [11], [12], in part mediated by insulin-like growth factors (IGF). In addition to the direct effect on growth regulation, GH has been shown to increase feeding behaviour in fish [11], however is not known whether this occurs by direct action on the central nervous system or indirectly through metabolic or downstream endocrine targets [12], [13]. It has been suggested that GH may pass through the blood-brain barrier and stimulate its own receptor in the CNS, affecting neuroendocrine secretion of appetite by regulating peptides such as neuropeptide Y, bombesin, and cholecystokinin [14].

Transgenic animals can be useful models for studying long-term functional effects of neuroendocrine systems without applying invasive procedures to the test organism. In this way, transgenic model organisms increase the possibility to examine the costs and benefits of increased production of hormones without treatment effects such as repeated hormone injections [15], [16]. Studying GH-transgenic organisms also provides an opportunity to investigate how changes in growth hormone axis alter fish development, growth, and morphometrics.

Growth hormone transgenic salmon constitutively expressing GH in non-pituitary tissues show elevated plasma GH levels, earlier fry emergence dates and increased daily specific growth rates compared to wild-type under hatchery-conditions [15], [17], [18], [19], [20], [21]. The significantly increased growth rate of GH transgenic fish is associated with strongly elevated appetite and feed intake, and feed conversion efficiency relative to wild-type [19], [21], [22], [23], [24]. Consequently, when fed to satiation, transgenic individuals of the same age as wild-type are much larger [17], [20]. GH-transgenic salmon also demonstrate greater general activity and, because of the increased drive to forage, are more willing to take risks [22], [25], [26], [27].

Many aquatic habitats are presently experiencing environmental shifts which may influence the rate of growth and maturation of animals, with consequences for fitness and further effects on ecosystems. Studying growth in different thermal conditions provides insights into the possible changes in and effects on physiological parameters (such as GH production and physiological effects) under changing ecological conditions. In the present study, the developmental rate and growth rates of wild-type and GH-transgenic coho salmon at 6 different temperatures ranging between 8° and 18°C has been assessed and modelled. Further, a morphometric analyses of juvenile salmon has been undertaken to detect differences in body shape of fish reared at different temperatures.

## Methods

### Experimental animals and conditions

This study was performed at the DFO/UBC Centre for Aquaculture and Environmental Research, West Vancouver, Canada which houses a contained aquatic system designed to prevent the escape of transgenic fish. Our research was approved by and conducted according to guidelines of the Department of Fisheries and Oceans Pacific Region Animal Care Committee (AUP 08-003). Coho salmon of wild genotype were the offspring of wild-caught parents from the Chehalis River, BC, Canada. Transgenic coho salmon were originally produced by microinjecting eggs from wild parents with the gene construct OnMTGH1 with a metallothionein-B promoter driving the over-expression of the type-I GH gene from sockeye salmon (*O. nerka*) [17]. The transgenic strain utilized (M77) was maintained through crosses with wild salmon and thus contain on average the same genetic background as the wild-type fish except for the presence of the OnMTGH1 transgene. Experimental transgenic fish were the offspring of wild caught females from the Chehalis River and homozygous M77 transgenic males reared at the experimental facility. Half-sib wild-type experimental fish originated from the same females that were crossed to wild males obtained from the Chehalis River.

### Hatching and development of eggs and alevin

In March 2006, 24 Whitlock-Vibert hatching boxes (www.fedflyfishers.org) were filled with 80 eyed eggs each of either transgenic or wild-type genotype (previously incubated at 10°C from fertilization). The boxes were placed into twelve 200 L tanks, which were divided into six groups. Fresh well water was either heated or chilled and mixed in a flow-through system to gradually change tank water temperatures from 10°C to constant 8°, 10°, 12°, 14°, 16° and 18°C (with 2 replicate tanks at each temperature). Temperatures of 8° and 12°C where changed over a period of 18 hours. 14°C, 16° and 18° C were reached after 28, 38 and 48 hours respectively. Two boxes, one with transgenic and the other with wild-type eggs were placed into each tank so both types experienced the same temperature change. Artificial light was kept on a 10 h light: 14 h dark photoperiod regime.

The hatching boxes were checked every second day and any dead eggs or alevin were recorded and removed. Dates for reaching specific stages such as hatching or complete absorption of the yolk sack were observed for each genotype and temperature.

### Growth of fry and juveniles

In May 2006, two floating incubation containers (15 L each, with bottom and side mesh to allow water flow), containing 30 first-feeding fry of either wild-type or transgenic genotype previously incubated at 10°C, were placed into each 200 L tank. The water temperatures were gradually changed to constant at 8°, 10°, 12°, 14°, 16° and 18°C (see above), the artificial daylight was constant at 10 h light:14 h dark per day. Thereafter, experimental fish were hand-fed to satiation, by throwing small amounts of pellets into the tank until fish lost interest in eating, with commercial fish food (Skretting Inc.,) from 6 (younger fry) to 2 (juveniles) times every day. The size of food pellets was chosen throughout the experiment to be appropriate according to fish size. Once every second week for 14 weeks, fish were either weighed in groups for an average group weight, or were weighed individually, resulting in seven data points for each group. After 14 weeks the replicate groups of the same genotype and temperature were pooled and released into the twelve 200 L tanks (genotypes separate) for an additional 35 days of growth.

### Growth/survival statistics and analyses

No significant differences were detected between the two replicates groups for the same treatment (at egg, fry or juvenile stages). Consequently, the data were pooled for further analyses.

The probability and time of egg hatching and survival of alevin to complete absorption of the yolk sack at different temperatures were tested with Kaplan-Meier survival analysis. Differences in alevin weight at the time when 50% of the individuals in a specific treatment group had absorbed their yolk sac completely, and data on the general weight differences between fry/juveniles of two genotypes at different temperatures, were tested with two-way ANOVAs with mass in grams as the dependent variable and the genotypes and temperatures as fixed factors.

The growth coefficient (Gc) for groups of juvenile coho salmon was calculated from a simple mathematical model presented by Iwama and Tautz [28], [29]. Gc was calculated based the following formula: W_f_^1/3^ =  W_i_^1/3^ + (T/1000× Gc) × Time; there W_f_^1/3^ is the cube root of the final weights in grams; W_i_^1/3^ is the cube root of the initial weight in grams; T is the temperature in °C and Time is in days between measurements of W_f_ and W_i_. A value 1 of Gc suggests that the fish are growing according to the model whereas values above or below indicate higher or lower growth rates, respectively, than what is predicted from the model. Additionally we fit our data into a classic exponential growth function: W =  a × e^b × time^, where *a* (intercept), and *b* (slope) were estimated constants.

### Morphometrics

**Figure 1:**
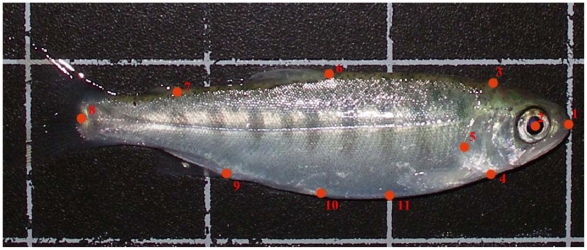
Locations of the 11 landmarks on a digital photograph. Red dots are marking the 1) tip of the nose, 2) centre of the eye, 3) dorsal dividing-line of head and body, 4) ventral dividing-line of head and body, 5) posterior point of operculum, 6) anterior end of dorsal fin, 7) anterior end of adipose fin, 8) central caudal dividing line of body and tail, 9) anterior end of anal fin, 10) anterior end of pelvic fin, 11) lowermost part of the stomach.

Differences in body shape among genotypes and rearing temperatures were examined by geometric morphometrics methods [30], [31]. We digitized 11 landmarks (Fig. 1) using the software program tpsDig2 [32]. Variation in shape was small enough to allow statistical analysis to be performed as assessed by TpsSmall [33]. Landmarks were analysed in Tps-Relw which uses the Generalized orthogonal least-squares Procrustes (GPA) procedure to produce both affine (uniform) and non-affine (non-uniform) partial warp scores (representing morphological deformations from a consensus individual) [34]. Differences in body shapes due to genotype and rearing temperature (while controlling for difference in size) were tested with a two-way MANCOVA where centroid size (similar to body size) was the covariate and the affine and non-affine partial warp scores as response variables (reported as Pillai's trace). Because the discriminant analysis used to describe groups differences was applied to the combined groups of genotype and temperature (hence n = 15 per group), partial warp scores could not be used. Instead, we extracted relative warps (RW) using TPSRelw [34]. An initial discriminant analysis was then applied to all relative warps and centroid size as predictor variables. The test of equality of group means was then used to assess which RW would be included in the final analysis since each group only had 15 individuals and hence not all 18 RW could be used for classification [35]. This strategy resulted in RW 3, 5, 8, 17, and 18 being excluded and the other 13 RW being included in subsequent analysis. This procedure allowed us to take advantage of the most information initially obtained from the partial warps scores considering both the number of landmarks and the sample size.

## Results

### Development

**Figure 2:**
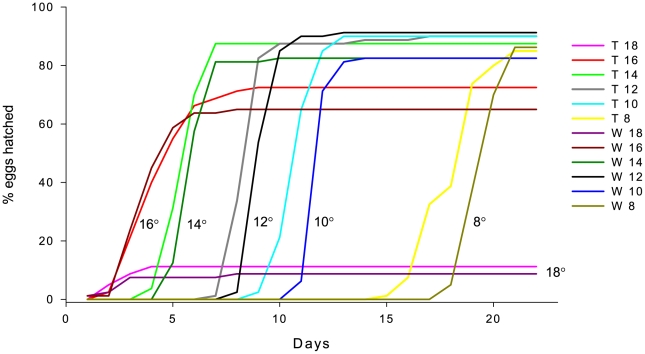
Timing and percentage of eggs hatching. Wild-type (W) and GH-transgenic (T) coho salmon eggs hatching at different temperatures (8–18°C).

There was an effect of both genotype and temperature on the probability and timing of hatching of coho salmon eggs (Fig. 2; left side of Table 1) with typically shorter time to hatch with increasing temperature, and transgenic genotypes hatching sooner than wild-type. At temperatures 8°, 10°, 12° and 14°C, a significant difference in hatching timing was found between wild-type and transgenic fish (χ^2^ = 10; 30; 11; 11 respectively for each temperature, p<0.01; 0.0001; 0.001; 0.001; Kaplan-Meier; pair-wise comparison Log Rank/Mantel-Cox). There was no difference in timing between genotypes at 16° and 18° C (χ^2^ = 0.23 and 0.28; p = 0.63; 60 respectively). The magnitude of difference in hatch timing between genotypes increased as temperature decreased.

**Table 1:** Temperature effects on hatching and survival.

	Hatch timing				Survival to first feeding			
	W		T		W		T	
°C	χ^2^	p	χ^2^	p	χ^2^	p	χ^2^	p
8 vs. 10	23.8	0.000	58.9	0.000	1.1	0.293	0.5	0.483
10 vs. 12	74.2	0.000	37.8	0.000	4.1	0.043	0.1	0.799
12 vs. 14	1.7	0.195	47.2	0.000	5.7	0.017	0.2	0.618
14 vs. 16	5.3	0.021	0.2	0.645	23.7	0.000	36.9	0.000
16 vs. 18	52.2	0.000	56.8	0.000	55.4	0.000	58.2	0.000

The table shows statistical output from comparison of temperature effects on the probability and timing of hatching (left side, compare also with Fig. 2), and on the survival to the absorption of the yolk sac (right side) of wild-type (W) and transgenic (T) coho salmon (Kaplan-Meier; Log Rank/Mantel-Cox).

**Figure 3:**
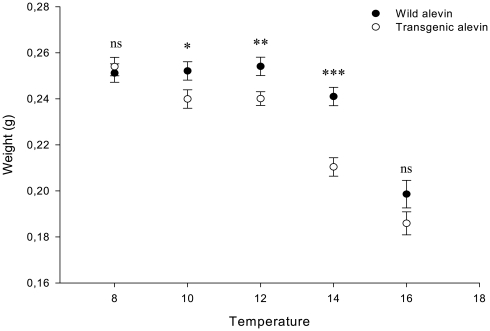
Average weights of alevin. Wild-type and GH-transgenic coho salmon alevin reared at different temperatures at the time when half of the group had completely absorbed their yolk sacks.

No differences in survival of fry (to the total absorption of the yolk sack stage) were found between different genotypes (p>0.05 at all cases; Kaplan-Meier; pair-ways comparison, Log Rank/Mantel-Cox). However we did observe differences in survival of alevin at different temperatures (right side of Table 1) with the maximum survival at 12 degrees (over 90% alive fry in both genotypes) and noticeably decreased survival values for fry at 16° (around 40% alive fry in both genotypes) and 18°C (2.5% live fry in both genotypes). The number of days from the date of hatching until the time when 50% of the group had absorbed their yolk sacks decreased with increasing temperature and differed between genotypes (from 35 days at 8°C to 25 days at 16°C in wild fish and from 28 days at 8° to 22 days at 16°C in transgenics). The number of day-degrees between hatching and buttoning up (closure of the abdomen) was higher at higher temperatures but lower in transgenics than in the wild fish (280 in wild alevin and 224 in transgenic alevin at 8°C; 400 in wild alevin and 352 in transgenic alevin at 16°C). There was a significant interaction effect between temperature and genotype on the weights of alevin at the time when 50% of the group had absorbed their yolk sacks (F_4.601_ = 5.1; p<0.001). There were no differences at 8 and 18 degrees, but transgenic alevin were found to be lighter than wild-type fish at intermediate temperatures (Fig. 3).

### Growth

**Figure 4:**
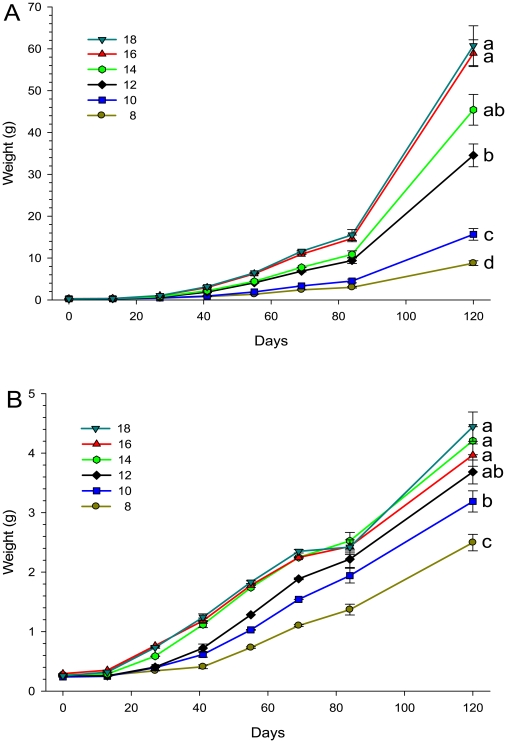
Growth of transgenic (A) and wild-type (B) coho salmon juveniles. Juveniles were reared from first-feeding until an age of 120 days at temperature from 8 to 18°C. Different letters denote significant differences at the p<0.05 level (Tukey's post hoc test) on ln-transformed values after 120 days (figures based on raw values). Note the more than 10-fold larger Y-axis scale for the transgenic fish.

Based on the size of fish at 120 days of growth post first feeding, there was a clear interaction between genotype and temperature, with transgenic fish growth being relatively more stimulated as temperature increased (Fig. 4). Since variances could not be made homogenous when analyzing the weight data from fry/juveniles (due to the large difference in growth between transgenic and wild-type fish), analyses of differences between temperatures were done separately for the two genotypes. For both transgenic (F_5, 269_ = 91.5, p<0.001) and wild-type (F_5, 265_ = 91.5, p<0.001) fish, temperature affected growth in a positive direction. In both genotypes, fish at 8°C grew slowest with an increasing temperature having a relatively larger effect on transgenic fish than on wild-type fish. The coefficient of variation for final weights was higher in transgenic than in wild-type at all temperatures (Table 2).

**Table 2:** Coefficient of variation (Cv) and growth coefficients (Gc).

	wild-type		transgenic	
T°C	Cv	Gc	Cv	Gc
8	0.38	0.77	0.47	2.96
10	0.37	0.82	0.57	4.16
12	0.31	0.79	0.46	7.94
14	0.40	0.78	0.54	8.96
16	0.34	0.64	0.39	10.18
18	0.36	0.65	0.54	9.34

The table shows the Cv of weight (Cv  =  standard deviation/average weight) at the end of the growth trial and Gc [29] for wild-type and transgenic coho salmon at different temperatures.

The percentage of juvenile coho salmon surviving to the day 120 of the experiment at 8° 10°, 12°, 14°, 16° and 18°C for wild-type fish were 78%, 73%, 55%, 85%, 88% and 72% respectively, and for transgenic fish 86%, 72%, 56%, 75%, 88% and 80% respectively. Tank effects are not suspected as effects were seen in all four tanks randomly distributed in the experimental design). Survival of the fish did not differ between genotypes (binary logistic regression; Wald χ^2^ = 0.12, p = 0.73) but did vary with temperature (Wald χ^2^ = 20.9, p<0.001) with no significant interaction (Wald χ^2^ = 4.3, p = 0.50). Because 16°C fish had the highest survival, this group was used as reference category to which survival of the others groups was compared (simple contrast coding in SPSS). This revealed that fish at 10°C (Wald χ^2^ = 4.2, p = 0.042), 12°C (χ^2^ = 14.5, p<0.001) and 18°C (χ^2^ = 4.9, p = 0.026) had lower survival than at 16°C with no difference between 16°C and 8°C or 14°C (both p>0.14).

**Figure 5:**
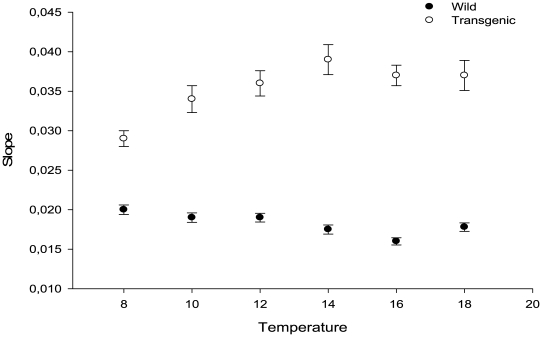
The slope (*b*) values of fitting the growth data to a classic exponential growth function: W =  a × e^b × time^. Error bars represent 95% confidence intervals.

In general the growth coefficient (Gc) values of the wild-type fish were found to be lower than predicted by the model of Iwama and Tautz [29], and further decreasing at higher temperatures. In contrast, the situation was the opposite in the transgenic fish (Table 2). Slopes estimated by the function W =  a × e^b × time^ are presented in Table 3 and Fig. 5. Transgenic salmon showed increasing growth rate slopes with temperature up to 14°C (Spearman r  = 0.81, P = 0.05), whereas wild-type fish showed a decreasing response to temperature (Spearman r  = −0.88, P = 0.020).

**Table 3:** Estimates of the growth function W =  a × e^b × time^.

Temp	Parameters	Transgenic			Wild-type		
		estimate	SE	R^2^	estimate	SE	R^2^
8	a	0.260	0.032	0.76	0.240	0.015	0.76
	b	0.029	0.001		0.020	0.001	
10	a	0.260	0.050	0.69	0.320	0.020	0.76
	b	0.034	0.002		0.019	0.001	
12	a	0.480	0.087	0.79	0.370	0.021	0.81
	b	0.036	0.002		0.019	0.001	
14	a	0.430	0.099	0.73	0.530	0.031	0.74
	b	0.039	0.002		0.018	0.001	
16	a	0.670	0.100	0.83	0.590	0.027	0.77
	b	0.037	0.001		0.016	0.000	
18	a	0.720	0.160	0.72	0.540	0.029	0.77
	b	0.037	0.002		0.018	0.001	

The table illustrates the estimates of intercept (*a*) and slope (*b*) and the estimate of the least-square fit to the function (R^2^). SE is based on the number of individuals.

### Morphometrics

Body shapes of fish were influenced by both centroid size (F_18, 150_ = 6.5, p<0.001) and interactions between genotype and temperature (F_90, 770_ = 6.5, p<0.001). Seven of 18 partial warps were significantly (p<0.05) affected by centroid size, nine were significantly different for genotype and nine for temperature, and six were significant for the interaction between genotype and temperature.

**Figure 6:**
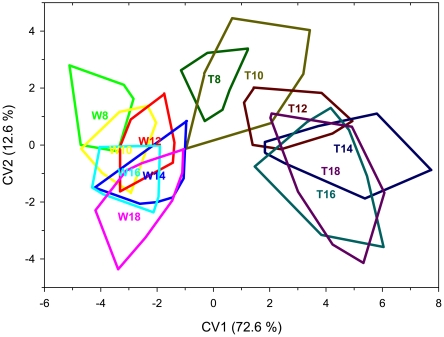
Canonic variate plot (CV). CV of transgenic (T) and wild-type (W) coho salmon body morphology reared at 8–18°C. Means of the populations in the discriminant space were significant between genotypes and groups except in wild-type fish at neighboring temperatures. and transgenic fish between 14–18°C.

The first discriminant function explained 72.6% and the second function explained 12.6% of the variation in shape. Means of the populations in the discriminant space were significantly different between all groups except the wild fish at 12° and 10°C and between wild fish 14° and 16°C, with no overlap between the genotypes. None of the wild fish at neighboring temperatures were significantly different after multiple test corrections, and transgenic fish from the 14–18°C groups were not significantly different. Separation of the groups was still high, with 97.8% of the fish being classified to the correct category (Fig. 6). Three wild fish were misclassified: one wild fish at 8° classified as a wild fish 10°C, one wild fish 16° as a wild fish 14°C, and one wild fish 18° as a wild fish 14°C. One 10° transgenic fish was misclassified as a 16° transgenic.

**Figure 7:**
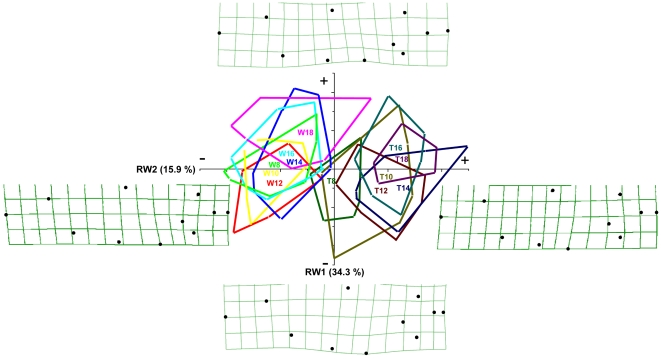
Relative warps. The plot shows the first two relative warps with corresponding deformation grid from the extreme of the relative warps axes. Derived from morphometric analyses of wild-type (W) and transgenic fish (T) reared at 8–18°C.

Visualization plots of the first two relative warps (RW) explaining together 50.2% of the variation in shape illustrates the clear difference between wild-type and transgenic genotypes (Fig. 7). Associated with negative values on the first RW were transgenic fish with relatively deeper bodies and smaller heads, especially the distance between the eye and the tip of the nose. Hence, this RW explained mostly deformations (change in landmark location) in vertical space. In wild-type fish, slender bodies are evident and relatively larger heads. Shape effects associated with RW2 were less clear but had a tendency to associate positive values with increasing temperature. The deformation grid suggests that most deformation for this RW was in horizontal space with positive values being associated with longer ventral and shorter dorsal arcs, with the opposite for negative values.

## Discussion

Defining the effects of temperature on biological functions is important to understand the contribution of climatic factors to the fitness and the ecological interactions of organisms at both individual and population levels [6]. Hormones play an essential role in the control of these mechanisms since many of them act on or are produced by the central nervous system and in that way link environmental stimuli to behaviours and physiology [36]. The present study shows a strong effect of temperature on developmental rate, juvenile growth and morphology that differed between wild-type and GH-transgenic coho salmon.

A positive correlation between growth rate and temperature is well known for most poikilotherm fish species, including salmonids [1]. Salmonid fishes show consistently increasing growth rates in temperatures ranging from 0°C to approximately 15°C [1], [37]. Within this interval, growth acceleration is achieved through higher metabolism supported by increased food consumption and conversion efficiency [6]. At very high temperatures, growth enhancement ultimately cannot be maintained because of rapidly ascending metabolic costs [1]. However, further increase in growth is still possible within a few degrees over the optimal, if demands for maintaining growth rate can be met by increased rations and sufficient oxygen supply. At very high temperatures (more than 20°C) the feeding rate of most salmonids declines sharply, and is completely inhibited at temperatures several degrees below the incipient lethal level. In this state, the non-optimal temperature limits the oxygen supply in the organism leading to hypoxemia and rapidly falling performance in fish [5].

Consistent with previous studies [37], we observed reduced egg and alevin survival rate at the highest study temperatures. At 18°C the percentage of eggs surviving to hatching averaged 9% for the two genotypes, but rapidly increased to 70% at 16°C and 90% at 12°C. Post-hatch alevin survival of both genotypes was highest at 12°C (ca 90% of hatchlings), whereas at 16°C less than 40% of alevin survived to complete absorption of the yolk sack.

GH and IGF-I are major regulators of somatic growth, and according to several studies, there is a close positive relationship between the levels of these hormones in fish and the ambient water temperature [38], [39], [40], [41], [42], [43], [44]. Both of these genes have been shown to be active in salmonid embryos, even before the development of the pituitary gland [43], [45], [46]. Yet, variation of temperature has been shown not to change (aside from timing arising from different rates of development) the levels of GH and IGF-I protein and transcript in salmonid embryos, implying that these hormones play a lesser role in mediating temperature effects during embryogenesis [43], [45], [46]. High produion of GH in salmon embryos in the present study, however, did, at lower temperatures (8° to 14°C), shorten the development time of eggs compared to wild-type [15], [20]. Thus, even though GH may not play a primary role in the regulation of temperature effect in salmon embryos, as evident in the present study, an over-expression of GH still promotes development and decrease the incubation time in transgenic coho embryos compared to the wild-type.

According to Gabillard et al. [39] the thermal influence on embryonic development of salmonids is best explained by the actions of another growth factor – IGF-II, as the amount of mRNA for this hormone does increase with temperature. IGF-II is known in mammals to act primarily as a mediator of growth prenatally [47]. The strain of GH transgenic salmon used in the present study does not display elevated levels of IGF-II mRNA at later stages of development (i.e. fingerling) [48], but it is not known whether this gene is activated by GH transgenesis at earlier stages. It is possible that the earlier hatching at higher temperatures (16° to 18°C) in both genotypes may have been mediated by temperature-mediated IGF-II action which was able to dominate over effects of GH. At later stages, GH transgenesis and temperature are both able to stimulate growth rate.

The temporal aspect of emergence of fry is a critical element in salmon life histories, and fry that emerge at appropriate times will have adaptive advantages in initial feeding and predator avoidance compared to the fry emerging too early or too late. Consequently, natural selection should time spawning events and egg development rates to correspond with the optimum time for fry emergence in each particular stream environment [49]. During the period of emergence the usage rate of yolk stores sustaining growth until the fry emerge depend strongly on environmental temperature [49], [50]. Fry that emerge at appropriate time will therefore have adaptive advantages in initial feeding, predator avoidance and in the temporal integration with other life history needs compared to the fry emerging too early or too late in the cycle.

Thermal conditions in nature fluctuate between years, creating slightly different temporal optima for each year in the spawning and incubation time. Fry with high production of GH will most likely hatch and emerge earlier than the fry with the lower expression of GH at all physiologically adaptive temperatures. This shift towards earlier hatching and emergence of eggs and fry of the GH-enhanced salmon may cause a fitness disadvantage in the life-histories of these fish at temperatures that are experienced by conspecifics under natural conditions. However, it is possible that being first to emerge could also confer an advantage for fry with high GH production, if they are able to establish territories before the conspecifics with lower GH production emerge [51]. The present data showed a greater decrease in body weight in transgenic alevin with increasing temperatures than in the wild-type fish (caused by a general increased usage rate of the yolk sack). Consequently, increased GH-production may lead to greater sensitivity to environmental conditions and an enhanced necessity to find sufficient food supplies compared to wild genotype [15]. Indeed, populations of GH transgenic fry are less able to withstand periods of limited food availability than are wild type populations [52].

Although the majority of the weight gain of anadromous salmonids occurs in the ocean environment, growth rate of juvenile fish in their home streams is also of great importance, especially for salmonids that must acquire minimum sizes before they can smolt and migrate to the sea. The size achieved by juvenile coho salmon at the end of their first summer has a strong effect on their later success of over-wintering and smolting. Larger size has also been shown to result in competitive advantages in processes of feeding and establishing territories [6]. As even minor temperature shifts strongly influence the growth and development in salmonids and cause essential alterations in life history patterns, any long-term non-adaptive variation in response to thermal conditions is likely to have important consequence on individual physiology and fitness [49].

GH secretion can be influenced by temperature [12], however, Danzmann et al. [53] found a reduced capacity of GH to influence growth of juvenile domesticated rainbow trout (*Oncorhyncus mykiss*) reared at high temperatures. In contrast, the present data showed a clear positive effect of temperature on growth rates of transgenic juvenile coho salmon, indicating that the over-production of GH does promote growth in this species even at high rearing temperatures. These two studies used different modes of administration of GH (injection, vs. endogenous overproduction in transgenics) which may result in differential rates of turnover of active GH protein that could influence growth stimulation effects. The cause for the low survival of coho salmon juveniles in this experiment at 12° is not known, however, it is possible that a pathogen has its peak of virulence at this temperature; however, this is only a speculation.

It is well-known that metabolic costs and requirements of oxygen of fish increase at higher temperatures, making increased growth rates difficult to maintain [1], [4]. The present study suggests that the increased growth rates of fish with elevated GH levels were supported at temperatures several degrees higher than in the wild-type fish. From the mathematical estimations of growth rates (Fig. 6) we observe an increase in the values of growth slopes of GH-enhanced salmon up to 14° C, whereas slopes were slightly negative over the entire range of temperatures in wild-type fish. In other words, the relationship between growth rate and relative size of fish is negatively affected by temperature in wild fish, but increases (up to 14° C) in fish with elevated GH-production. Thus, over-production of GH in coho salmon allows them to meet the increasing demands of growth by enhanced nutritional intake to a larger degree than occurs in wild-type, suggesting that the optimal thermal conditions for GH-enhanced coho salmon might be higher than for the wild-type fish.

Iwama and Tautz [29] developed a simple model for predicting the growth of salmonids at different temperatures in intensive aquaculture conditions. Their model includes assumptions (such as growth increasing steadily with increasing temperature) that may not be true under all circumstances, and thus should be applied with this knowledge in mind [54]. However, their model is still useful to compare growth slopes of groups of salmonids (for instance between strains with different growth rates, or populations in different environmental conditions). In the present experiment we calculated growth coefficients (Gc), comparing the actual growth rate represented by our data with the theoretical growth rate of the model. Both genotypes showed best agreement with the model at lower temperatures but reacted to increased temperatures in opposite ways: wild-type fish showed a lower than predicted growth rate at higher temperatures, whereas the Gc values for GH-transgenic fish were up to 10 times higher at high temperatures than predicted by the model. These relative differences in Gc between genotypes at different temperatures suggest a greater response to temperature in fish with elevated GH levels than predicted by the model. When comparing our results to the Gc values for various stocks of salmonids (see Table 1 in Iwama [28]) we find that the Gc values of the wild-type fish in general agree with data from other salmonids, whereas the Gc for GH-enhanced fish differs greatly (as predicted from their known accelerated growth).

Different rearing temperatures not only affected the growth rate but also the body morphology of juvenile wild-type and GH-enhanced coho salmon. While the change in body shape in wild-type fish seemed to be gradual over the temperatures, in GH-enhanced salmon the effect was sharper, giving rise to a group of fish (reared at 14°–18°C) characterized by very large bodies and relatively small heads. Earlier studies analyzing the morphological effects of GH-transgenesis in fish have demonstrated noticeable differences in the shape and development of the head and in most cases also a change in the head/body size ratio compared to the wild fish [55], [56], [57], [58]. In accordance with these morphological changes, fish with increased GH-production in this study in general showed relatively deeper bodies and smaller heads with a reduced distance between the eye and the tip of the nose, whereas wild-type fish were characterized by slender bodies and relatively larger heads. However a tendency to longer ventral and shorter dorsal arcs was noticed at higher temperatures in both genotypes.

Changes in body shape could affect the swimming capacity of fish and change their ability to escape predators [59]. A lower swimming speed of fast-growing GH-transgenic coho salmon of the same size as wild-type has been observed [60]. A genotype effect on body shape was evident in the present study which could influence swimming capacity in transgenic fish compared to wild-type, but it could also reduce susceptibility to predators, particularly when these are gape-limited [61]. Thus it is conceivable that GH-levels in fish in natural surroundings would be heavily exposed to balancing natural selection modifying the rates of somatic growth. The direction of the selection will likely depend on multiple factors such as abundance and type of predators and food availability.

The present study has observed effects of temperature on growth, survival and body morphology in GH transgenic and wild type coho salmon. Importantly, the two genotypes respond differently to temperature, indicating genotype by environment interactions are influencing phenotypic development between these strains.
